# Comparison of an AI-assisted system (S-detect) and sonographers of different experience levels in diagnosing thyroid nodules: a retrospective study

**DOI:** 10.3389/fonc.2025.1656919

**Published:** 2025-10-06

**Authors:** Zhenhao Zheng, Yang Yu, Jun Li, Ting Ma, Wen Liu

**Affiliations:** The First Affiliated Hospital of Shihezi University, Shihezi, China

**Keywords:** S-detect, artificial intelligence, ultrasound, thyroid nodules, AI

## Abstract

**Background:**

Thyroid cancer (TC), the most common neck malignancy, can metastasize early. Conventional ultrasound diagnosis relies on subjective feature interpretation. Objective tools are needed to improve diagnostic efficiency. Objective: To compare the diagnostic efficacy of artificial intelligence-assisted ultrasonography (S-Detect) versus sonographers of varying experience in differentiating benign from malignant thyroid nodules.

**Methods:**

This retrospective study analyzed 315 thyroid nodules (237 patients) undergoing ultrasound and biopsy/surgical confirmation. Sonographers were classified as junior or advanced. The diagnostic performance (sensitivity, specificity, accuracy, kappa, Youden’s Index, AUC) of S-Detect and both sonographer groups was compared.

**Results:**

In the junior group (115 nodules), S-Detect outperformed junior sonographers (sensitivity 98.4% vs 96.9%, specificity 78.4%vs 52.9%, accuracy 89.6% vs 77.4%, kappa 0.784 vs 0.521, AUC 0.884 vs 0.749; all P<0.05) In the advanced group (200 nodules), S-Detect sensitivity (97.5%) matched senior sonographers (96.7%), but with lower diagnosis specificity (57.7% vs 69.2%). Senior sonographers showed higher accuracy (86.0% vs 82.0%) and kappa (0.691 vs 0.593), Compared with senior physicians, S-Detect demonstrated comparable diagnostic efficacy to the senior group in identifying malignant nodules, while showing slightly inferior performance to senior ultrasound specialists in diagnosing benign nodules. Senior physicians exhibited superior accuracy and consistency in nodule diagnosis compared to S-Detect; however, no significant difference was observed between the two in overall performance (P > 0.05).

**Conclusion:**

S-Detect surpasses junior sonographers in diagnosing thyroid nodules. Its overall diagnostic performance is comparable to advanced sonographers.

Thyroid cancer, the most common malignant tumor of the endocrine system, has shown a sustained and significant upward trend in incidence worldwide, including in China. According to data from the 2023 China Cancer Report, thyroid cancer ranks seventh among new cancer cases in China and fourth among new cancer cases in women (1).

Two-dimensional ultrasound (US) is the preferred method for routine examination of thyroid nodules, with an accuracy rate of 67%-92% for diagnosing benign and malignant thyroid nodules (2), However, ultrasound images of thyroid nodules are complex and diverse, and certain ultrasound features of benign and malignant nodules overlap, leading to phenomena such as “same image, different disease” (i.e., similar ultrasound findings but different postoperative pathological diagnoses) or “same disease, different image” (i.e., the same pathological type presenting different ultrasound findings in different patients).Although multiple studies have confirmed that ultrasound features such as hypoechoic, solid. irregular margins, aspect ratio >l, and microcalcifications are highly associated with thyroid malignant tumors, relying solely on a single or combination of multiple features remains insufficient for a comprehensive and integrated assessment of nodule benignity or malignancy in clinical practice. Studies indicate that approximately 20% of thyroid nodule diagnoses are challenging when relying solely on conventional ultrasound examinations (3).

S-Detect technology is a software system based on computer-aided diagnosis (CAD) (4). Its core principle involves utilizing convolutional neural networks (CNN) to perform deep learning and training on a large number of ultrasound images with histopathological results, thereby constructing a diagnostic model. The workflow of the S-Detect system primarily consists of three key steps: (l) lesion segmentation (2); ultrasound feature description (3); benign/malignant differentiation assistance (5), The system integrates risk stratification criteria from multiple authoritative guidelines, including those from the Korean Thyroid Association (KTA), the American Thyroid Association (ATA), and the American College of Radiology (ACR). By quantitatively analyzing ultrasound gray-scale parameters, the system analyzes the morphology, margins, posterior echoes, internal echoes, and calcifications of thyroid nodules in the acquired images. Ultimately, S-Detect generates a binary classification diagnosis result of “likely benign” or “likely malignant” and provides a structured report to serve as a reference for the final diagnosis by ultrasound physicians (5), S-Detect technology helps improve the efficiency of radiologists in distinguishing between benign and malignant thyroid and breast nodules, reduces their workload, and optimizes clinical workflows, making it one of the most commonly used Al-assisted systems in this field (6).

Although previous studies have compared the diagnostic efficacy of artificial intelligence with that of sonographers, this research aims to stratify sonographers according to their diagnostic experience. It will investigate the differences in efficacy between the S-Detect-assisted diagnostic system and sonographers of varying proficiency levels in distinguishing benign from malignant thyroid nodules. This will further analyse the value of artificial intelligence in clinical ultrasound practice.

## 1 Subjects and methods

### 1.1 Study population

A retrospective review was conducted of 364 thyroid nodules from280 patients who underwent FNAB or surgical resection at the First Affiliated Hospital of Shihezi University between December 2022 and June 2023. All patients underwent US examination prior to surgery, and S-Detect was performed during the ultrasound examination. and pathological results were obtained postoperatively. The study underwent rigorous sample size calculations, which were not mentioned in the text. The sample size calculation process is presented here for your review.

According to literature review, the sensitivity of S-Detect technology in diagnosing benign versus malignant thyroid nodules is 84.35%, with a specificity of 86.76%. The estimated sensitivity of this method is 84.35%, and the estimated specificity is 86.76%, with a permissible error margin between 0.05 and 0.1. Calculate the sample size according to the formula: *n=(z_α_/δ)^2^·(1-p)·p*

(P denotes the sensitivity or specificity of the screening method under evaluation)

(1) When the permissible error is 0.05, the sample sizes for the two groups are:

Case group n_1_ = (1.96/0.05) ² × (1 – 0.8435) × 0.8435 = 199.8 ≈ 200

Control group n_2_ = (1.96/0.05) ² × (1 – 0.8676) × 0.8676 = 176.7 ≈ 178

(2) When the permissible error = 0.1, the sample sizes for both groups are:

Case group n_1_ = (1.96/0.1) ² × (1 – 0.8435) × 0.8435 = 49.9 ≈ 50

Control group n_2_ = (1.96/0.1) ² × (1 – 0.8676) × 0.8676 = 44.2 ≈ 45

The total sample size for both groups in this project, calculated using the above formula, ranges between 95 and 378.

#### 1.1.1 Inclusion criteria

(1) Patients scheduled for FNAB or thyroid mass resection at our hospital (2); New cases (3); Patients who had not taken oral levothyroxine, propylthiouracil or methimazole prior to surgery and had not received radioactive ^131^l therapy.

#### 1.1.2 Exclusion criteria

**Figure 1:**
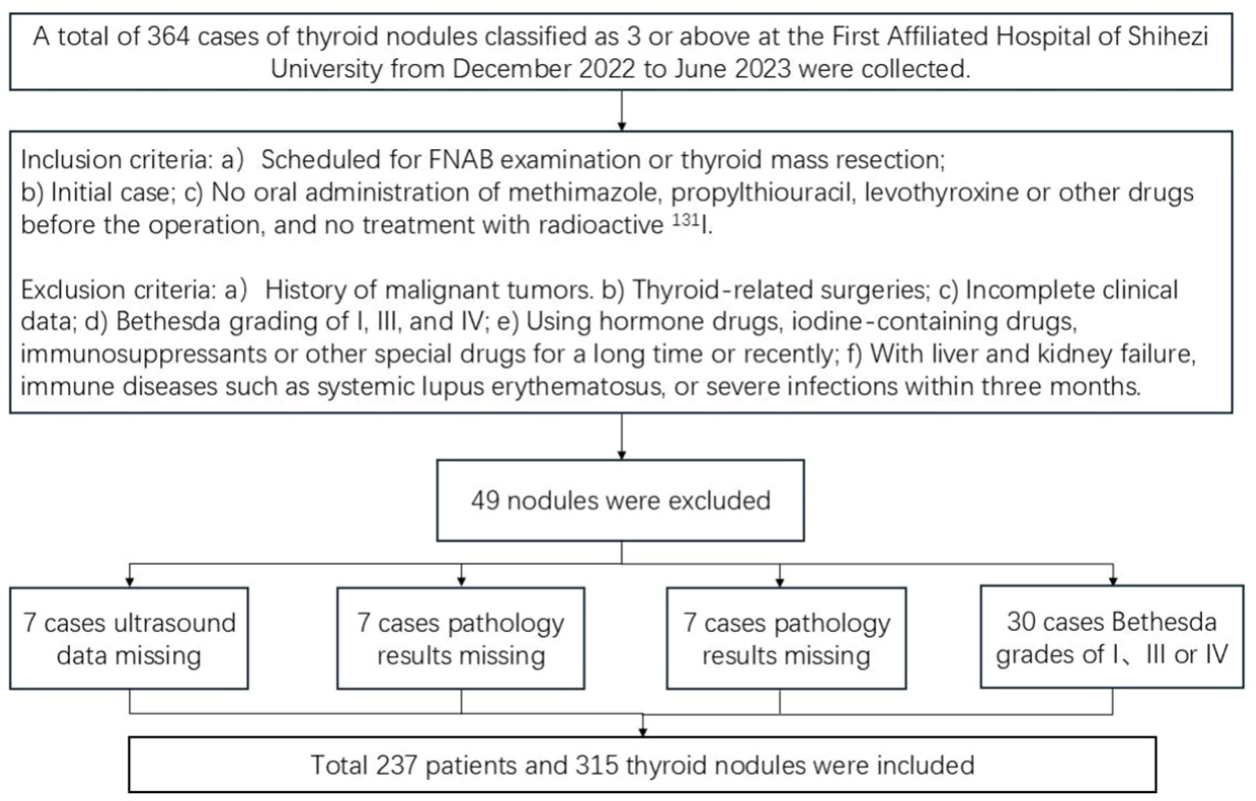
Flowchart of case inclusion and exclusion.

(l) Patients with a history of malignant tumors (2); Patients who have previously undergone thyroid-related surgery (3); Patients with incomplete clinical data(e.g., lacking thyroid two-dimensional ultrasound examination results, pathological diagnosis results, etc.) (4); Patients with Bethesda grading of I, III, or IV after FNAB (5); Patients who have been taking or using topical steroid medications, iodine-containing medications, immunosuppressants, or other special medications for an extended period or recently (6);Patients with liver or kidney failure, immune disorders such as systemic lupus erythematosus(SLE), or severe infections within the past three months. A total of 315 nodules meeting the criteria were ultimately included (**Figure 1**).

### 1.2 Ultrasound physician grading

Diagnostic physicians with the title of attending physician or below are defined as the junior group, while diagnostic physicians with the title of associate chief physician or above are defined as the senior group.

### 1.3 Routine ultrasound examination

Samsung R10 color Doppler ultrasound diagnostic instrument is used. The patient is instructed to lie flat on the examination bed with the head slightly tilted backward to fully expose the neck, breathe calmly, apply an adequate amount of coupling agent, and select the L3-12A linear array probe with a frequency of 3–12 MHz. Enter the thyroid scanning mode, with the probe close to the skin without applying pressure. First, perform routine transverse and longitudinal section scans of the overall thyroid structure, then focus on suspicious thyroid nodules, collecting and recording the following ultrasound characteristics: Nodule location (right lobe, left lobe, or isthmus), number (single/multiple),size (cm x cm), internal structure (cystic, cystic-solid, spongy, solid), echogenicity(hypoechoic, extremely hypoechoic, anechoic, hyperechoic, isoechoic), aspect ratio (>1, = 1.<1), calcification (no calcification, microcalcification, coarse calcification, or coarse calcification with microcalcification), margin (regular or irregular), border (clear or unclear)glandular invasion (present or absent), and blood flow (no blood flow within, minimal blood flow, peripheral blood flow, or abundant blood flow), etc. Subsequently, ultrasound physicians of various seniority levels used the ACR TI-RADS system to classify suspicious nodules, defining nodules classified as ACR TI-RADS category 4 or higher as malignant and those below category 4 as benign.

### 1.4 S-detect examination

**Figure 2:**
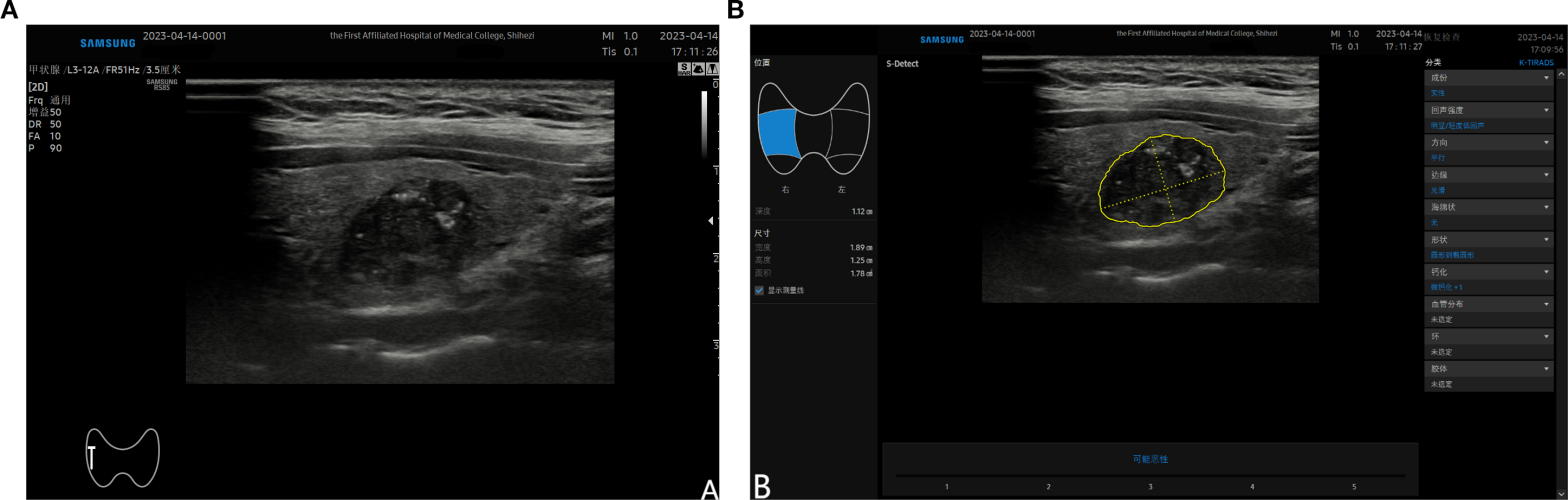
**(A)** shows a malignant nodule located in the mid-section of the right lobe of the thyroid detected by ultrasound; **(B)** demonstrates the automatically outlined nodule area after entering the S-Detect mode and selecting the “auto tracing” option. The nodule characteristics are displayed on the right side of the screen, and the analysis results are shown at the bottom.

After completing the routine ultrasound examination, switchback to the standard two-dimensional ultrasound mode. Adjust the probe position to ensure the target nodule is clearly visible, aiming to achieve the maximum diameter of the nodule and a cross-section perpendicular to it, while clearly displaying the relationship between the nodule and surrounding tissues. Press the freeze button, fine-tune the trackball until the image is clear, enter S-Detect mode, and ensure the sampling box fully encompasses the target nodule. The program will automatically outline the nodule region; manually adjust or fine-tune by clicking if necessary. Finally, the program automatically analyzes the target region and provides an analysis result of “possibly malignant” or “possibly benign”, along with a structured report for reference. (**Figure 2**). This S-Detect examination was performed by a physician with over eight years’ experience in ultrasound, who is proficient in S-Detect operation techniques and was not involved in the conventional ultrasound imaging of the nodule previously. Throughout both the conventional ultrasound and S-Detect examinations, all clinicians remained blinded to the nodules’ final pathological findings. Furthermore, the clinician responsible for conventional ultrasound imaging and the clinician responsible for the S-Detect examination were mutually unaware of each other’s results.

### 1.5 Pathological examination

The patient’s postoperative pathology or FNAB results are provided in a standardized pathology report issued by a pathologist with more than five years of experience, FNAB results are graded according to the 2017 Bethesda Thyroid Cytopathology Reporting System (7), with grading criteria shown in (**Table 1**). Postoperative thyroid pathology is based on the final pathological paraffin-embedded section as the gold standard. If a patient with a nodule undergoes only FNAB without nodule excision for histopathological assessment, Bethesda Grade V-VI is considered malignant; Bethesda Grade II, with nodule stability or volume reduction observed via ultrasound follow-up, is considered benign (8), Among the 217 patients with 315 nodules, 173 patients underwent FNAB, involving227 nodules, of which 59 nodules were classified as Bethesda Grade II and did not undergo further nodule excision surgery; 165 patients underwent thyroid nodule excision surgery, involving 217 nodules.

**Table 1:** FNAB grading standards.

Bethesda grading	Diagnosis results
I	Unable to diagnose/Unsatisfactory
II	Benign lesion
III	Cell atypia of undetermined significance/Follicular lesion of undetermined significance
IV	Follicular tumor/Suspected follicular tumor
V	Suspicious malignant tumor
VI	Confirmed as malignant thyroid tumor

### 1.6 Statistical analysis

Statistical analysis was performed using SPSS 26.0. Quantitative data are expressed as (x ± s). FNAB results or postoperative pathology were used as the gold standard to analyze the diagnostic performance of S-Detect and the two groups of diagnostic physicians in distinguishing benign from malignant thyroid nodules. The chi-square test was used to compare the sensitivity, specificity, and accuracy of S-Detect and the two groups of ultrasound diagnostic physicians, with a significance level of o = 0.05. The Kappa test was used to evaluate the consistency of results among S-Detect, ultrasound physicians, and different groups of ultrasound physicians. When the Kappa value was >0.8, it indicated high consistency of results; when the Kappa value was 0.6-0.8, it suggested moderate consistency of results; When the Kappa value is between 0.4 and 0.6, it indicates moderate consistency; when the Kappa value is less than 0.4, it indicates low consistency, requiring further analysis. ROC curves were constructed for S-Detect and ultrasound physicians, and the area under the curve (AUC) was calculated. The Delong test was used for comparison. A P-value less than 0.05 was considered statistically significant.

## 2 Results

### 2.1 Grouping of junior and senior physicians

The junior group comprised 115 nodules, while the senior group contained 200 nodules: within the junior group, 37 patients presented with two nodules in the thyroid, and 41 patients had one nodule. These 41 nodules were labelled as ‘difficult’ by the junior physicians. Subsequently, all 115 nodules from these 78 patients underwent secondary assessment by senior physicians, who were unaware of the primary physicians’ initial diagnoses. The senior physicians independently assessed 85 nodules from 81 patients, with no involvement of the primary physicians in this process. Therefore, in the study design, considering that both the primary and senior groups assessed the same nodules in some overlapping patients (115 nodules from 78 patients), this common subset was incorporated as two separate samples. This ultimately resulted in the inclusion of 315 nodules from 237 patients.

### 2.2 Pathological findings

A total of 315 nodules from 237 patients were included, comprising 80 males and 235 females, with a mean age of 50.3 ± 11.73 years. Among these, 129 nodules (41.0%) received a final pathological diagnosis of benign nodule, with an age distribution of 52.4 ± 11.43 years; 186 nodules (59.0%) were diagnosed as malignant, with an age distribution of 48.8 ± 11.74 years.

### 2.3 Diagnostic performance analysis of the junior group

**Figure 3:**
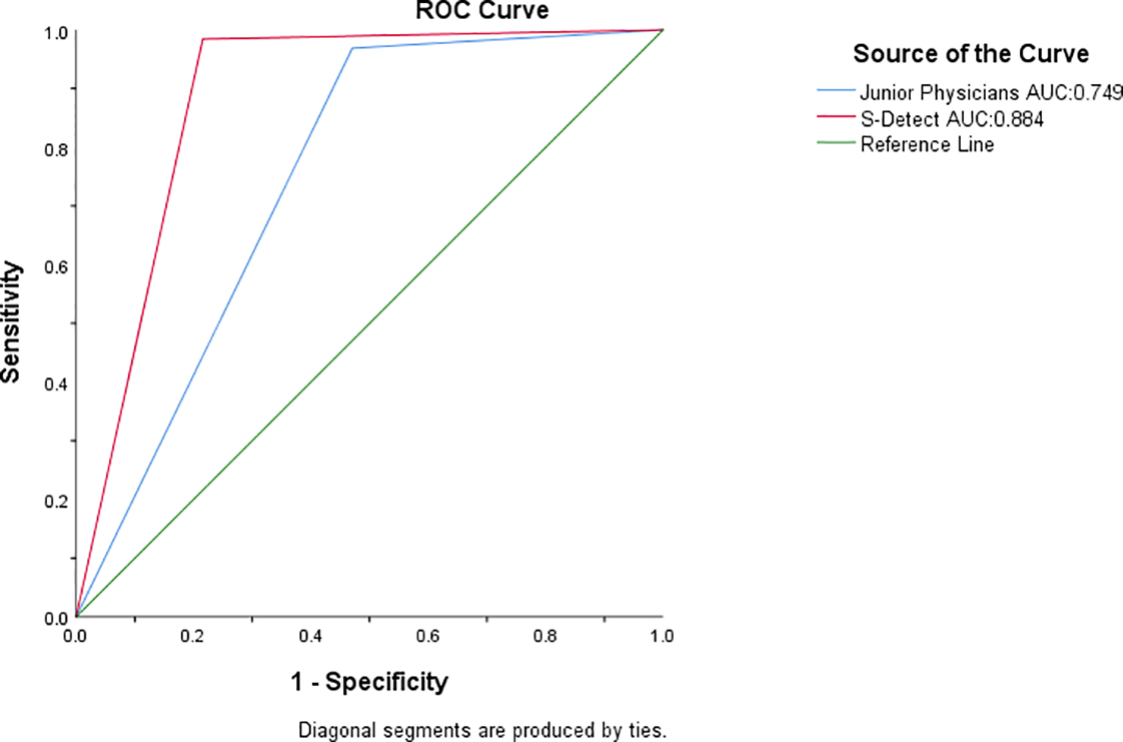
AUC of the junior physicians and S-detect. The green line is the reference line, the blue line is the junior physician Group, and the red line is S-detect.

Among the 115 nodules, 5l were benign and 64 were malignant. The analysis results of the 115 thyroid nodules by S-Detect and junior group diagnostic physicians are shown in (**Table 2**) and (**Table 3**), and the AUC curve results are shown in (**Figure 3**). Among them, S-Detect had a diagnostic sensitivity of 98.4%, specificity of 78.4%, accuracy of 89.6%, and AUC of0.884 (P< 0.01) in the junior group; The junior physicians had a diagnostic sensitivity of 96.9%, specificity of 52.9%, accuracy of 77.4%, and an AUC of 0.749 (P< 0.01) in the junior group.

**Table 2:** S-Detect diagnostic performance in the junior group.

S-Detect	Pathological diagnosis		Sensitivity	Specificity	Accuracy	χ^2^	*P*	Youden	Kappa
	Malignant	Benign							
Malignant	63	11	98.4%	78.4%	89.6%	73.10	0.000	0.769	0.784
Benign	1	40							

**Table 3:** Junior physician diagnostic performance in the junior group.

Junior Physician	Pathological diagnosis		Sensitivity	Specificity	Accuracy	χ^2^	*P*	Youden	Kappa
	Malignant	Benign							
malignant	62	24	96.9%	52.9%	77.4%	37.35	0.000	0.753	0.521
benign	2	27							

### 2.4 Diagnostic performance analysis of the senior group

**Figure 4:**
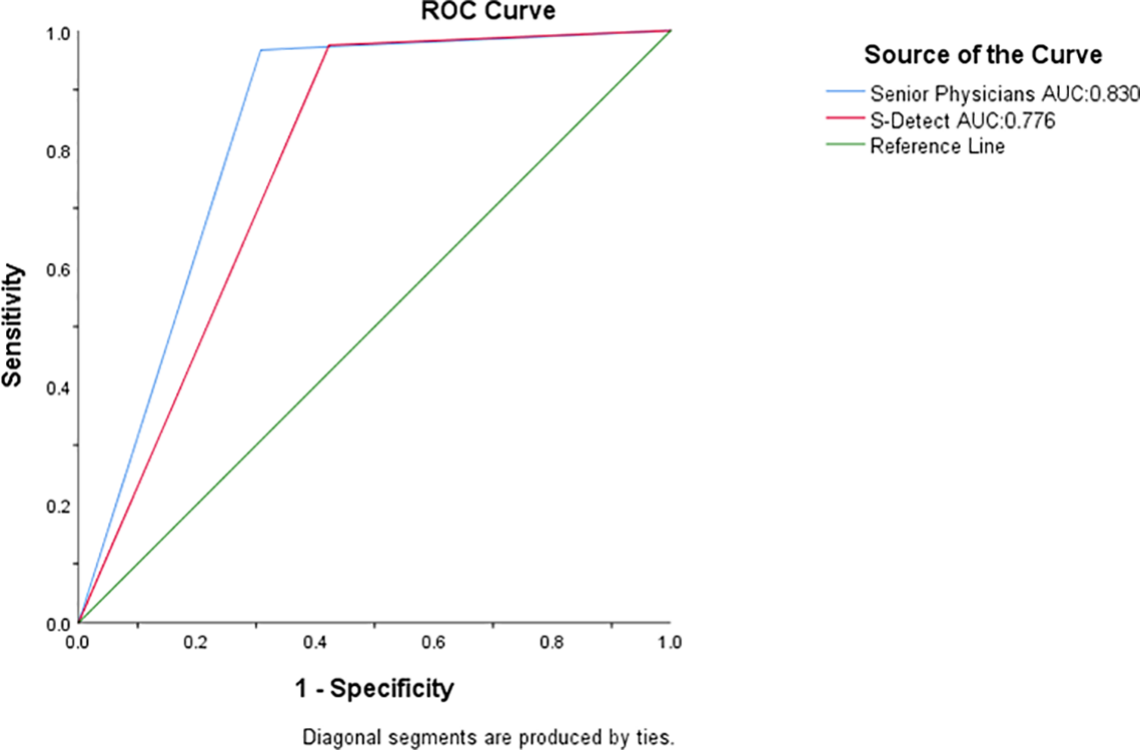
AUC of the senior physician and S-Detect. The green line is the reference line, the blue line is the senior physician group, and the red line is S-detect.

Among the 200 nodules, 78 were benign, and 122 were malignant. The analysis results of the 200 thyroid nodules by S-Detect and the senior group diagnostic physicians are shown in (**Table 4**) and (**Table 5**), and the AUC curve results are shown in (**Figure 4**). Among them, S-Detect had a diagnostic sensitivity of 97.5%, specificity of 57.7%, and accuracy of 82.0% in the senior group, with an AUC of0.776 (P <0.01); the senior physicians had a diagnostic sensitivity of 96.7%, specificity of 69.2%, and accuracy of86% in the senior group, with an AUC of 0.830 (P<0.01).

**Table 4:** S-Detect diagnostic performance in the senior group.

S-Detect	Pathological diagnosis		Sensitivity	Specificity	Accuracy	χ^2^	*P*	Youden	Kappa
	Malignant	Benign							
Malignant	119	33	97.5%	57.7%	82.0%	79.58	0.000	0.550	0.593
Benign	3	45							

**Table 5:** Senior physician diagnostic performance in the senior group.

Senior Physician	Pathological diagnosis		Sensitivity	Specificity	Accuracy	χ^2^	*P*	Youden	Kappa
	Malignant	Benign							
Malignant	118	24	96.7%	69.2%	86.0%	100.51	0.000	0.660	0.691
Benign	4	54							

#### 2.4.1 Comparison between S-detect and junior physicians

In the junior group, a chi-square test was performed to compare the sensitivity and specificity of S-Detect with those of junior physicians’ diagnoses, yielding a chi-square value of 37.51, P< 0.05 (**Table 6**). A DeLong test was also conducted to compare the AUC values of the two, revealing statistical significance with P = 0.001<0.01. The difference was statistically significant. (**Table 6**).

**Table 6:** S-Detect compares with junior physician.

Inspection method	Sensitivity	Specificity	χ^2^	*P*	AUC difference	Standard deviation	95% CI	z	*P*
S-Detect	98.4%	78.4%	37.51	0.000	0.135 3	0.0415	0.054 ~ 0.217	3.2587	0.0011
Jonior Physician	96.9%	52.9%							

#### 2.4.2 Comparison of S-detect with senior physicians

In the senior group, a chi-square test was performed on the sensitivity and specificity of S-Detect compared with the diagnostic results of senior physicians, yielding a chi-square value of 64.91, P < 0.05 (**Table 7**). The AUC values of the two were also subjected to a Delong test, which detected significance with P = 0.129 >0.05, there was no statistically significant difference (**Table 7**).

**Table 7:** S-Detect compares with senior physician.

Inspection method	Sensitivity	Specificity	χ^2^	*P*	AUC difference	Standard deviation	95% CI	z	*P*
S-Detect	97.5%	57.7%	64.91	0.000	0.053 6	0.035 3	-0.016 ~ 0.123	1.516 8	0.129 3
Senior Physician	96.7%	69.2%							

## 3 Discussion

Ultrasound examination has become the preferred imaging modality for thyroid nodules due to its non-invasive, convenient, and real-time dynamic imaging advantages (9), With the widespread use of screening and the refinement of diagnostic criteria (such as the ACR TI-RADS system (10), the detection rate of thyroid nodules has increased significantly (11). However, ultrasound diagnosis highly depends on the operator’s experience, and diagnostic consistency varies across different levels of medical institutions: diagnostic accuracy in high. volume medical centers may be affected by workload, while resource-limited regions are constrained by physician experience and equipment conditions (12), The development of artificial intelligence technology offers a new approach to address these limitations (13, 14). CAD systems like S-Detect use deep learning to standardize the analysis of thyroid nodule features (15).

This study compared the diagnostic efficacy of an artificial intelligence-assisted diagnostic system (S-Detect) with that of ultrasound practitioners of varying levels of expertise in distinguishing benign from malignant thyroid nodules. Key findings are as follows: Significant value in assisting junior physicians: In the junior group (n=115), S-Detect demonstrated significantly higher diagnostic sensitivity (98.4% vs 96.9%), specificity (78.4% vs. 52.9%), and AUC (0.884 vs. 0.749) were significantly superior to those of junior physicians (all P< 0.01), and diagnostic consistency was higher (Kappa value 0.784 vs.0.521).

In comparison with the diagnostic efficacy of the senior physicians: The sensitivity of S-Detect technology in diagnosing benign versus malignant nodules is comparable to that of senior-level ultrasound practitioners, whilst its specificity is marginally lower than that of senior-level practitioners, with statistically significant differences observed (sensitivity 97.5% vs 96.7%, specificity:57.7% vs 69.2%, P = 0.000), but slightly lower AUC (0.776 vs 0.830, P = 0.129>0.05), with no statistically differences. The diagnostic consistency of S-Detect was also slightly lower than that of senior physicians (Kappa value 0.593 vs 0.691). This indicates that S-Detect is comparable to senior physicians in terms of detecting malignant nodules (sensitivity 97.5% vs 96.7%), but senior physicians demonstrate superiority in distinguishing benign nodules (specificity 69.2% vs 57.7%). However, there is no significant difference in overall performance between S-Detect and senior physicians.

This study shares some consistency with previous reports: Choi (16) found that the sensitivity of S-Detect was comparable to that of senior physicians (90.7% vs. 88.4%), but its specificity was lower (74.6% vs. 94.9%); Wei (17) confirmed its diagnostic enhancement value for junior physicians. Of particular note is that the specificity and AUC values for both the S-Detect and physician groups in this study were lower than those reported in some literature, primarily due to the following reasons (1): Case selection bias: All included nodules were ACR TI-RADS category 4 or higher lesions (including a small number of category 3 nodules but >2.5 cm in size) scheduled for FNAB or surgical resection. Such nodules often exhibit malignant ultrasound features, leading to an increased false-positive rate in the benign group (2) Inherent characteristics of the diagnostic system: The Kwak TI-RADS system, on which S-Detect is based, inherently exhibits a tendency toward high sensitivity and low specificity (18), further exacerbating the risk of false positives. Therefore, the findings presented by this study demonstrate the diagnostic efficacy of junior physicians, senior physicians, and S-Detect when encountering thyroid nodules with a high malignant risk in real clinical practice.

This study has the following limitations (1): The single-center design may limit the representativeness of the sample (2),S-Detect relies on static image analysis and cannot utilize the advantages of real-time dynamic ultrasound (19), which may affect the accuracy of feature interpretation (3); Clinical information (such as inflammatory history) was not fully integrated, only patients who underwent FNAB or thyroidectomy were included, and the sample size for negative nodules was not expanded, leading to some false-positive misclassifications. Future studies should focus on multicenter validation, optimization of dynamic image analysis algorithms, and multimodal clinical imaging fusion to further enhance diagnostic specificity. Additionally, future studies should include thyroid nodules diagnosed as benign through multiple examinations and regular follow-up, albeit without pathological confirmation, and involve both clinicians and S-Detect in diagnostic analysis to comprehensively evaluate diagnostic efficacy across different groups.

## 4 Conclusion

S-Detect can significantly improve the diagnostic ability of junior physicians in detecting thyroid nodules. Its ability to detect malignant nodules is comparable to that of senior physicians, and its ability to distinguish benign nodules is slightly lower compared to senior physicians. Although it has limitations in terms of’ specificity, it is suitable as a standardized auxiliary tool, especially in primary care settings. Through the combination of multi-modal diagnostic methods such as elastic imaging and contrast imaging, it is expected to establish a more accurate thyroid nodule stratification management system.

## Data Availability

The original contributions presented in the study are included in the article/supplementary material. Further inquiries can be directed to the corresponding author.
